# P-1016. Real World Utilization of Live-brpk (VOWST) among Patients with Recurrent Clostridioides difficile Infection: Single Center Experience

**DOI:** 10.1093/ofid/ofaf695.1212

**Published:** 2026-01-11

**Authors:** Drew Siskin, Nirja Mehta, Colleen S Kraft, Leah Wray

**Affiliations:** Emory University, Atlanta, Georgia; Emory University, Atlanta, Georgia; Emory University, Atlanta, Georgia; Emory Healthcare, Atlanta, Georgia

## Abstract

**Background:**

Recurrent *Clostridioides difficile* infection (rCDI) is facilitated by the depletion of key bacterial taxa in the gut microbiome. Live-brpk is an oral therapy of encapsulated Firmicutes spores administered following antibiotics for *Clostridioides difficile* to prevent rCDI. Since live-brpk was FDA approved in 2023, there are few reports of the outcomes of this therapy outside of clinical trials, which excluded several high-risk groups, including immunocompromised patients.

**Methods:**

We evaluated outcomes in adults referred for rCDI to the Infectious Diseases Clinic at Emory University. Patients were given a choice between live-brpk, live-jslm, and while available, fecal microbiota transplantation and bezlotoxumab. Patient demographic and clinical information was obtained through interview and chart review. Renal disease was defined as chronic kidney disease Stage 3b or higher. Immunocompromise was defined by use of immunosuppressants, typically for organ transplant or rheumatologic conditions. rCDI following live-brpk was defined as a positive *Clostridioides difficile* PCR/EIA which was treated with antibiotics.

**Results:**

Twenty-two individuals received live-brpk for the treatment of recurrent CDI; 55% were female with a median age of 69 years (range 22-89). 60% of patients had renal disease, 36% of patients were immunocompromised, including four patients with a history of solid organ transplantation, and one with recent stem cell transplantation. 45% of patients had greater than five lifetime episodes of CDI. No patients had recurrence of CDI within eight weeks after receiving live-brpk. Five patients recurred within six months, one of these patients was immunocompromised. Three patients received a second dose of live-brpk and remained free from recurrence for at least eight weeks. No patients reported side effects for which they required medical care.

**Conclusion:**

Among patients with high lifetime incidence of rCDI, following live-brpk >70% of patients did not have CDI recurrence six months after therapy. Patients with immunocompromise were treated safely in this cohort and had a similar recurrence rate as non-immunocompromised patients.

**Disclosures:**

Colleen S. Kraft, MD, MSc, Rebiotix Ferring: Advisor/Consultant|Vedanta: Advisor/Consultant

